# Enhancing malaria detection in resource-limited areas: A high-performance colorimetric LAMP assay for *Plasmodium falciparum* screening

**DOI:** 10.1371/journal.pone.0298087

**Published:** 2024-02-09

**Authors:** Tuyet Kha Nguyen, Hojong Jun, Johnsy Mary Louis, Ernest Mazigo, Wang-Jong Lee, Hyun Cher Youm, Jieun Shin, Douglas K. Lungu, Creto Kanyemba, Md Atique Ahmed, Fauzi Muh, Se Jin Lee, Sunghun Na, Wanjoo Chun, Won Sun Park, Joo Hwan No, Min-Jae Kim, Eun-Taek Han, Jin-Hee Han

**Affiliations:** 1 Department of Medical Environmental Biology and Tropical Medicine, School of Medicine, Kangwon National University, Chuncheon, Gangwon-do, Republic of Korea; 2 Noul Co., Ltd., Yongin, Gyeonggi-do, Republic of Korea; 3 Wezi Medical Centre, Mzuzu, Malawi; 4 ICMR-Regional Medical Research Centre, NER, Dibrugarh, Assam, India; 5 Faculty of Public Health, Department of Epidemiology and Tropical Diseases, Universitas Diponegoro, Semarang, Indonesia; 6 Department of Obstetrics and Gynecology, Kangwon National University Hospital, Chuncheon, Gangwon-do, Republic of Korea; 7 Department of Pharmacology, School of Medicine, Kangwon National University, Chuncheon, Gangwon-do, Republic of Korea; 8 Department of Physiology, School of Medicine, Kangwon National University, Chuncheon, Gangwon-do, Republic of Korea; 9 Host-Parasite Research Laboratory, Institut Pasteur Korea, Seongnam, Gyeonggi-do, Republic of Korea; 10 Department of Infectious Diseases, Asan Medical Center, University of Ulsan College of Medicine, Seoul, Republic of Korea; University of Health and Allied Sciences, GHANA

## Abstract

Malaria eradication efforts in resource-limited areas require a rapid, economical, and accurate tool for detecting of the low parasitemia. The malaria rapid diagnostic test (mRDT) is the most suitable for on-site detection of the deadliest form of malaria, *Plasmodium falciparum*. However, the deletions of *histidine rich protein 2* and *3* genes are known to compromise the effectiveness of mRDT. One of the approaches that have been explored intensively for on-site diagnostics is the loop-mediated isothermal amplification (LAMP). LAMP is a one-step amplification that allows the detection of *Plasmodium* species in less than an hour. Thus, this study aims to present a new primer set to enhance the performance of a colorimetric LAMP (cLAMP) for field application. The primer binding regions were selected within the A-type of *P*. *falciparum 18S rRNA* genes, which presents a dual gene locus in the genome. The test result of the newly designed primer indicates that the optimal reaction condition for cLAMP was 30 minutes incubation at 65°C, a shorter incubation time compared to previous LAMP detection methods that typically takes 45 to 60 minutes. The limit of detection (LoD) for the cLAMP using our designed primers and laboratory-grown *P*. *falciparum* (3D7) was estimated to be 0.21 parasites/μL which was 1,000-fold higher than referencing primers. Under optimal reaction condition, the new primer sets showed the sensitivity (100%, 95% CI: 80.49–100%) and specificity (100%, 95% CI: 94.64–100%) with 100% (95% CI: 95.70–100%) accuracy on the detection of dried blood spots from Malawi (*n* = 84). Briefly, the newly designed primer set for *P*. *falciparum* detection exhibited high sensitivity and specificity compared to referenced primers. One great advantage of this tool is its ability to be detected by the naked eye, enhancing field approaches. Thus, this tool has the potential to be effective for accurate early parasite detection in resource-limited endemic areas.

## Introduction

Malaria is a life-threatening infectious disease caused by *Plasmodium* species parasites and transmitted through the bites of infected female *Anopheles* mosquitoes. Early diagnosis is the most critical factor for malaria control and elimination as it actively interrupts transmission and prompts early treatment when patients have relatively mild symptoms. In 2021, malaria incidence was estimated at 247 million cases, with 619,000 malarial deaths in 84 endemic countries [1]. Moreover, over the past two years, the COVID-19 pandemic has hampered malaria control programs, leading to 13.4 million additional malaria cases [1]. Consequently, there is a growing concern for consistent malaria control and early diagnosis.

Since 2010, the World Health Organization (WHO) have been recommending microscopy and rapid diagnostic tests (RDTs) for diagnosis of malaria especially in resource-limited areas to ensure prompt diagnosis and treatment [2]. However, both microscopy and RDTs are prone to error in the reading of final result and exhibit low sensitivity at the low parasitemia compared to molecular-based detection tools [3,4]. Depending on the skills of microscopists, the quality of the reagents, and monitoring systems, microscopy detection has an estimated limit of detection (LoD) exceeding 50–500 parasites/μL in patient whole blood. [3,5]. Although RDTs demonstrate sensitivity around ~100 parasites/μL and high specificity by capturing parasite-specific antigens such as histidine-rich protein 2 (HRP2) for *P*. *falciparum*, the long half-life of HRP2 in patient blood circulation after parasite clearance can lead to false positive results [6–8]. Additionally, the sensitivity of the RDTs is reduced by *pfhrp2* and *pfhrp3* gene deletions, which leads to false negatives. A meta-analysis has highlighted a global pooled prevalence of 21.3% for *pfhrp2* gene deletions and 34.5% for *pfhrp3* deletions [9]. Moreover, the false negatives resulting from *pfhrp2* and *pfhrp3* deletions has been reported as 41.1% globally, predominantly observed in South America and subsequently in African regions [9]. To overcome this limitation, several molecular detection methods have been developed for diagnosis of malaria, including nested polymerase chain reaction (PCR), quantitative PCR, PCR-restriction fragment length polymorphism, multiplex PCR, and droplet digital PCR (ddPCR) [10–12]. At the present, PCR is the most sensitive method available, however, its high cost, time-consuming, and a requirement of a well-equipped laboratory limit the field application [13,14]. Thus, novel isothermal amplification techniques such as the isothermal recombinase polymerase amplification (RPA) and loop-mediated isothermal amplification (LAMP) has been intensively developed [15,16].

The LAMP principle hinges on the action of *Bacillus stearothermophilus* (*Bst*) DNA polymerase, enabling isothermal amplification through the processes of auto-cycling strand displacement and self-sustained sequence replication. This polymerase is activated at 60°C to 65°C and produces a large quantity of target-specific amplicons within 30 to 60 minutes. The set of four specific primers recognizes six different regions within the target gene sequence, resulting in high specificity and amplify 10^10^ replications of stem-loop structures [17]. Although conventional LAMP offers certain advantages, including ease of application in the field and higher sensitivity compared to PCR, it encounters limitations when incorporating amplicon indicators [18–20]. For instance, the use of polyethyleneimine has been found to hinder amplification efficiency, and ethidium bromide has been associated with potential mutagenic, carcinogenic, or teratogenic risks. Moreover, the process of introducing detection reagents or dyes poses a risk of cross-contamination. Therefore, colorimetric LAMP (cLAMP) has been developed as a promising molecular method for detecting various infectious diseases [21–23]. Numerous LAMP assays have been employed for the diagnosis of *P*. *falciparum*, targeting conserved genes such as *pfhrp2* and mitochondrial DNA [24–27]. However, previous studies had detection limits, ranging from 1 to 5 parasites/μL, with incubation times of 40 to 60 minutes [27–29]. To enhance the sensitivity and specificity for the early detection of *P*. *falciparum*, we developed and demonstrated a newly designed primer set for cLAMP targeting the A-type of *18S rRNA* gene located at chromosome 5 and 7 [30]. Then, we applied PCR-based diagnosis methods and real-time cLAMP to evaluate the sensitivity and specificity of the new primer set. The sensitivity and specificity of the designed primer set were compared with the referenced primer set described elsewhere and tested on the extracted clinical samples from Malawi [15].

## Materials and methods

### Colorimetric LAMP (cLAMP) primers design

Human malaria species *18S rRNA* genes were searched on NCBI (https://www.ncbi.nlm.nih.gov/) and PlasmoDB (https://plasmodb.org/plasmo/app) for the design of *P*. *falciparum* specific primers. The obtained sequences were aligned using the CLUSTAL-W algorithm of MegAlign software (DNASTAR, Madison, WI) **(S1 Fig)**. The phylogenetic tree was generated using the maximum likelihood method with 1,000 bootstrap pseudo-replicates for accuracy assessment in MEGA11 software [31].

**Fig 1:**
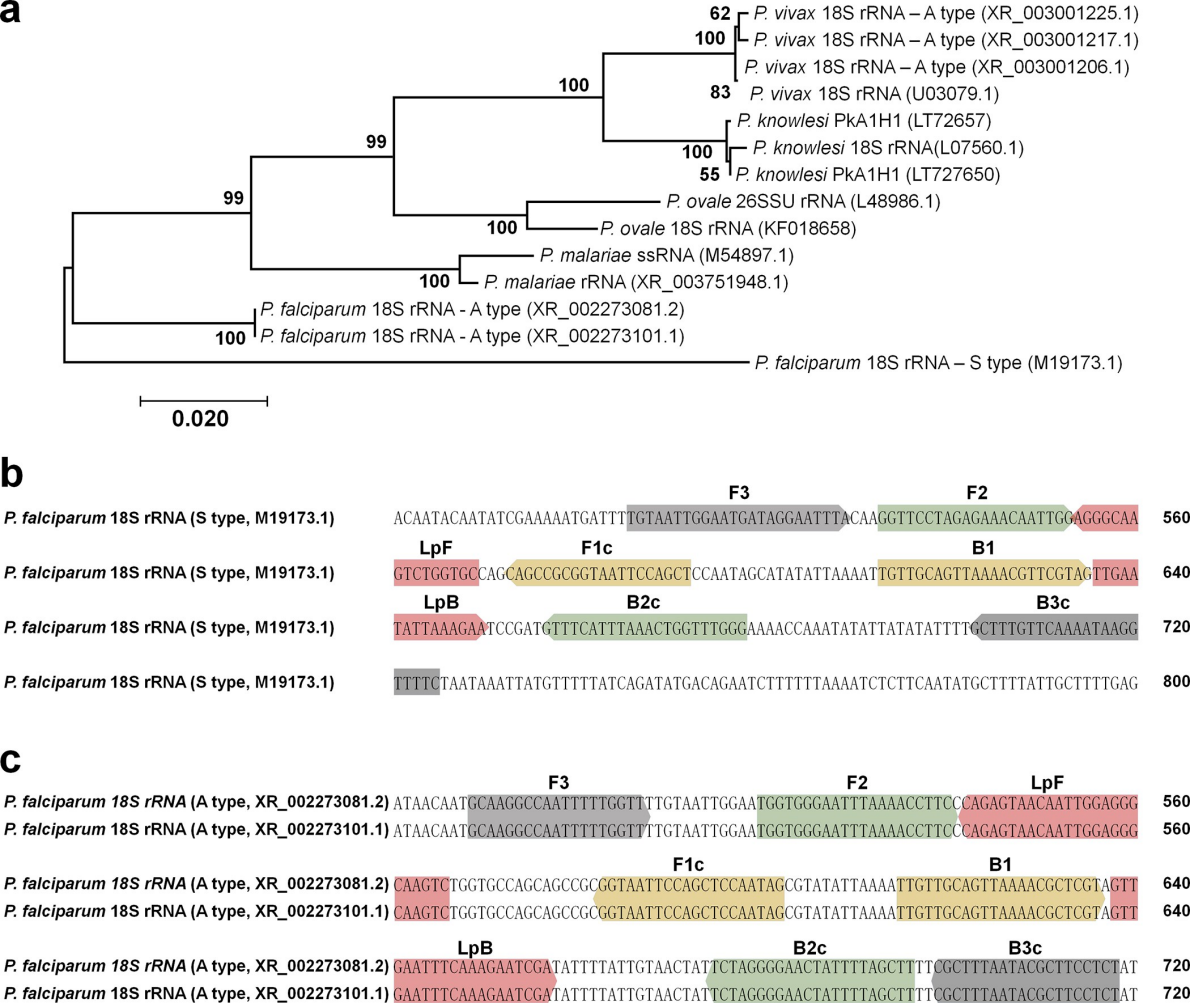
The phylogenetic tree of the *Plasmodium 18S rRNA* gene and the primer binding regions for cLAMP detection. **(a)** A maximum-likelihood phylogenetic tree of the *18S rRNA* gene from five *Plasmodium* species, supported by bootstrap from 1,000 pseudo replications. **(b)** The reference primer binding regions are used for LAMP detection. **(c)** The primer binding regions for designed primer in this study targeted the A-type of *18S rRNA* genes located on chromosome 5 (XR_002273101.1) and 7 (XR_002273081.2).

The initial set of four specific primers (F3/B3c and FIP (F1c-F2)/BIP (B1-B2c)) with six binding regions, along with two loop primers (LpB/LpF), were designed using Primer Explorer v5 (Eiken Chemical, Tokyo, Japan) (http://primerexplorer.jp/lampv5e/index.html). The final set of cLAMP primers was manually modified based on the criteria for designing LAMP primers (https://loopamp.eiken.co.jp/en/) and was subsequently verified for specificity using primer-BLAST on NCBI **(Table 1 and Fig 1C)**.

**Table 1:** Primer sequence for cLAMP detection of *P*. *falciparum 18S rRNA* gene.

Primer set	Primer	Sequence (5’-3’)
**Referenced primer** ^**a**^	F3	TGTAATTGGAATGATAGGAATTTA
	B3c	GAAAACCTTATTTTGAACAAAGC
	FIP (F1c-F2)	AGCTGGAATTACCGCGGCTG GGTTCCTAGAGAAACAATTGG
	BIP (B1-B2c)	TGTTGCAGTTAAAACGTTCGTAG CCCAAACCAGTTTAAATGAAAC
	LpF	GCACCAGACTTGCCCT
	LpB	TTGAATATTAAAGAA
**Designed primer**	F3	GCAAGGCCAATTTTTGGTT
	B3c	AGAGGAAGCGTATTAAAGCG
	FIP (F1c-F2)	CTATTGGAGCTGGAATTACC TGGTGGGAATTTAAAACCTTC
	BIP (B1-B2c)	TTGTTGCAGTTAAAACGCTCGT AAGCTAAAATAGTTCCCCTAGA
	LpF	GACTTGCCCTCCAATTGTTACTCTG
	LpB	GTTGAATTTCAAAGAATCGA

^a^ LAMP reference primer has been described by Han et al. (2007) [15].

### Plasmid DNA construction

Plasmid DNA (pDNA) was used to verify the cLAMP primer specificity within *Plasmodium* species and to determine the limit of detection (LoD). The partial nucleic acid sequences of the *18S rRNA* genes from five human malaria parasites were synthesized and cloned into the pTWIST-Amp pDNA (Twist Bioscience, CA) **(S1 Table)**. The pDNA was initially transformed into DH5-alpha competent *E*. *coli* cells using the heat shock method and subsequently cultured in 5 mL of LB broth containing 100 mg/mL ampicillin. Then, the pDNA was purified using a Plasmid DNA extraction kit (Macherey-Nagel, Duren, Germany), following the manufacturer’s protocol. The concentration of the extracted pDNA was measured using a spectrometer (Titertek-Berthold, Pforzheim, Germany) and then subjected to ten-fold serial dilution for further analysis, including primer specificity and sensitivity studies.

### *P*. *falciparum* genomic DNA preparation

To determine the LoD based on the parasite count, the *P*. *falciparum* 3D7 strain (ATCC) was cultured using human erythrocytes (2% hematocrit) in RPMI-1640 medium (Invitrogen, CA). The medium was supplemented with 2.3 g/L sodium bicarbonate, 0.05 g/L hypoxanthine, 10% Albumax I solution, and 10 mg/mL gentamicin. The culture was maintained in a gas mixture of 90% N_2_, 5% O_2_, and 5% CO_2_ and incubated at 37°C. Thin blood films were prepared following standard protocols, and parasitemia was measured through Giemsa staining under a microscope at 100X magnification. When the parasitemia reached 5%, genomic DNA was extracted from the cultured samples using a QIAamp DNA mini kit (QIAGEN, Hilden, Germany), following the manufacturer’s protocol. The genomic DNA (gDNA) was utilized for both confirming the LoD analysis by serial dilution of gDNA and verifying the performance of real-time colorimetric LAMP (RT-cLAMP).

### Colorimetric LAMP setting

The cLAMP reaction mixture was prepared in a total volume of 25 μL, comprising 12.5 μL of WarmStart® Colorimetric 2X Master Mix (NEB, MA), 0.96 μM of each outer primer (F3 and B3c), 7.68 μM of each inner primer (FIP and BIP), 3.84 μM of each loop primer (LpF and LpB), 1.0 μL of template DNA (pDNA or gDNA), and 5.5 μL of double-distilled water (DDW). The incubation was carried out using the ProFlex PCR System (Applied Biosystems, MA), and the optimal conditions for the cLAMP to detect *P*. *falciparum* were determined at 65°C for 30 minutes. Finally, the amplification products were confirmed directly by naked eye observation and electrophoresis with 2% agarose gel.

### Polymerase Chain Reaction (PCR)

PCR amplification was performed to confirm the species specificity and sensitivity of the outer primer set (F3-B3c) **(S2 Table)**. PCR mixture was prepared in a total volume of 20 μL comprising AccuPower PCR Premix (Bioneer, Daejeon, Republic of Korea), 1 μL of template DNA (pDNA or gDNA), 16 μL of DDW, and 1.0 μL of each 10 μM outer primers. The PCR condition consisted of an initial denaturation step at 95°C for 5 minutes, followed by 35 cycles (95°C for 30 seconds, 56°C for 30 seconds, and 72°C for 30 seconds), and a final extension step at 72°C for 5 minutes. The amplicons were visualized using electrophoresis with 2% agarose gel.

### Nested PCR

To diagnose *P*. *falciparum* in field isolates, we employed nested PCR (nPCR) to compare with cLAMP results [10]. The nPCR mixture was prepared in a total volume of 20 μL, which included AccuPower PCR Premix (Bioneer), 1 μL of template DNA (gDNA), 16 μL of DDW, and 1.0 μL of each 10 μM primer. The initial PCR reaction utilized genus-specific primers (rPLU1 and rPLU5) to amplify the *18S rRNA* gene **(S2 Table)**. DNA was amplified following conditions: 95°C for 10 minutes, 35 cycles (95°C for 30 seconds, 56°C for 30 seconds, and 72°C for 1.5 minutes), and a final extension at 72°C for 10 minutes. In the second PCR reaction, 1 μL of the first PCR product was combined with *P*. *falciparum*-specific primers (rFAL1 and rFAL2) and subjected to a final extension at 72°C for 30 seconds **(S2 Table)**. The amplicons were visualized using electrophoresis with 2% agarose gel.

### Quantitative PCR

Quantitative PCR (qPCR) was performed and compared to verify cLAMP performance using cultured *P*. *falciparum* and field isolates [11]. Reactions were conducted in a total volume of 20 μL, including 10 μL of 2X PrimeTime Gene Expression Master Mix with reference dye (Integrated DNA Technologies, IA), 4 μL of template DNA (gDNA), 10 μM species-specific primers, and 5 μM of a TaqMan probe **(S2 Table)**. The TaqMan probe assay was performed on an AriaMx Real-Time PCR System (Agilent Technologies, CA) with the following cycling conditions: hot-start polymerase activation at 95°C for 10 min, followed by 40 cycles of denaturation at 95°C for 15 seconds and final annealing for 65 seconds at 65°C.

### Real-time colorimetric LAMP (RT-cLAMP)

We adapted the cLAMP assay to verify its real-time performance. This real-time cLAMP (RT-cLAMP) was conducted by adding SYBR Green I fluorescence (Invitrogen, CA) into the cLAMP reaction, and signals were acquired every minute using the AriaMx Real-Time PCR System (Agilent Technologies). The cLAMP reaction mixture was prepared as described above, with the addition of SYBR Green I (1:10,000) for visualizing amplicon amounts and ROX (1:250) as a reference dye. The incubation was carried out at 65°C for 60 minutes, and the reaction data were analyzed using the Agilent AriaMx software (Agilent Technologies).

### Diagnosis of clinical isolates and ethical approval

Dried blood spots (DBS) from field isolates were collected during a clinical evaluation study of a malaria diagnostic device called miLab™ (Noul Co., Yongin, Republic of Korea). The clinical isolates were confirmed to have *P*. *falciparum* infection without other species co-infection using Bioline MALARIA Ag *P*.*f* (Abbott, IL) and microscopy during the collection of isolates. Regardless of their age, informed consent was obtained from patients seeking treatments at Mzuzu Urban Health Center and who were suspected to have malaria infections. All consent were drawn prior to participation, and for children parents/guardians were consented for their involvement. The study received approval on June 6, 2021, from the National Health Sciences Research Committee (NHSRC), a department of the Ministry of Health (MoH) in Malawi (IRB00003905). The samples were collected between June 2021 and May 2022. Clinical isolates were confirmed through microscopic examination at the Mzuzu Urban Health Centre by a trained technician. Whole blood samples were spotted onto Whatman 903 protein saver cards (Whatman International Ltd., Maidstone, England) and allowed to air-dry completely during collection, enclosed into separate plastic bags and transported to Kangwon National University for further molecular analysis. A total of eighty-four dried blood spots (DBS) were randomly selected from the collected samples and used in this study. Genomic DNA was extracted following the manufacturer’s protocol for QIAamp DNA Mini Kits (QIAGEN). The extracted gDNA was subsequently used for nested PCR (nPCR), quantitative PCR (qPCR), and colorimetric LAMP (cLAMP) to compare their performance for molecular diagnosis [10,11]. Parasitemia in the clinical isolates ranged from 1,069 to 285,290 parasites/μL in total 16 samples out of 84 samples, as observed through microscopic examination. All molecular diagnosis were conducted blindly, without prior knowledge of the microscopic examination results. The cLAMP reaction’s endpoint product was visualized with the naked eye under optimal conditions at 65°C for 30 minutes of incubation. All experiments using human specimens adhered to relevant guidelines and regulations and were approved on May 24, 2023, by the Kangwon National University, Republic of Korea (IRB No. KWNUIRB-2023-05-008).

### Data visualization and statistical analysis

The qPCR and RT-cLAMP data were obtained from Agilent AriaMx software (Agilent Technologies), and graph visualized by GraphPad Prism 8 software. Comparison of parasite count (parasites/μL) versus qPCR cycles or cLAMP incubation time (min.) and linear regression calculation were visualized by SigmaPlot software v12.

The statistical calculations of clinical specimen diagnosis were performed using the MEDCALP statistical software, which is accessible at https://www.medcalc.org/calc/diagnostic_test.php. Data were compiled into double-entry tables for calculation of sensitivity, specificity, and positive and negative predictive values for each test, along with the determination of 95% confidence intervals.

## Results

### Colorimetric LAMP primer design

We conducted an analysis of phylogenetic relationships among human malaria parasites to identify the colorimetric LAMP (cLAMP) primer. The A-type *18S rRNA* gene sequences of *Plasmodium* species formed phylogenetic clusters with its respective orthologs **(Fig 1A)**. Thus, the A-type of *18S rRNA* gene is suitable for designing a cLAMP primer set with conserved sequences at the intra-species level. We selected the most cited primer set for LAMP assay from previous studies and used as the reference primer set **(Fig 1B and Table 1)** [15]. This reference primer targeted the *P*. *falciparum* S-type *18S rRNA* gene, whereas the newly designed primer set targeted the A-type *18S rRNA* gene. The A-type *18S rRNA* gene exhibits a high copy number (1 x 10^4^ copies per ring stage parasite) at the asexual stage and shares an identical nucleic acid sequence on chromosomes 5 (XR_002273101.1) and 7 (XR_002273081.2) **(Fig 1C and Table 1)**.

### Specificity of cLAMP primer between *Plasmodium* species

To validate the specificity of the colorimetric LAMP (cLAMP) primers, we synthesized partial *18S rRNA* gene fragments from human malaria species **(S1 Table)**. The S-type and A-type of *P*. *falciparum 18S rRNA* genes (PfS and PfA) were used to verify the reference primer set and the designed primer set, respectively [15]. Furthermore, homologous regions of the A-type *18S rRNA* gene in its orthologs, including *P*. *vivax* (PvA), *P*. *knowlesi* (PkA), *P*. *malariae* (PmA), and *P*. *ovale* (PoA), were synthesized to confirm primer specificity.

**Fig 2:**
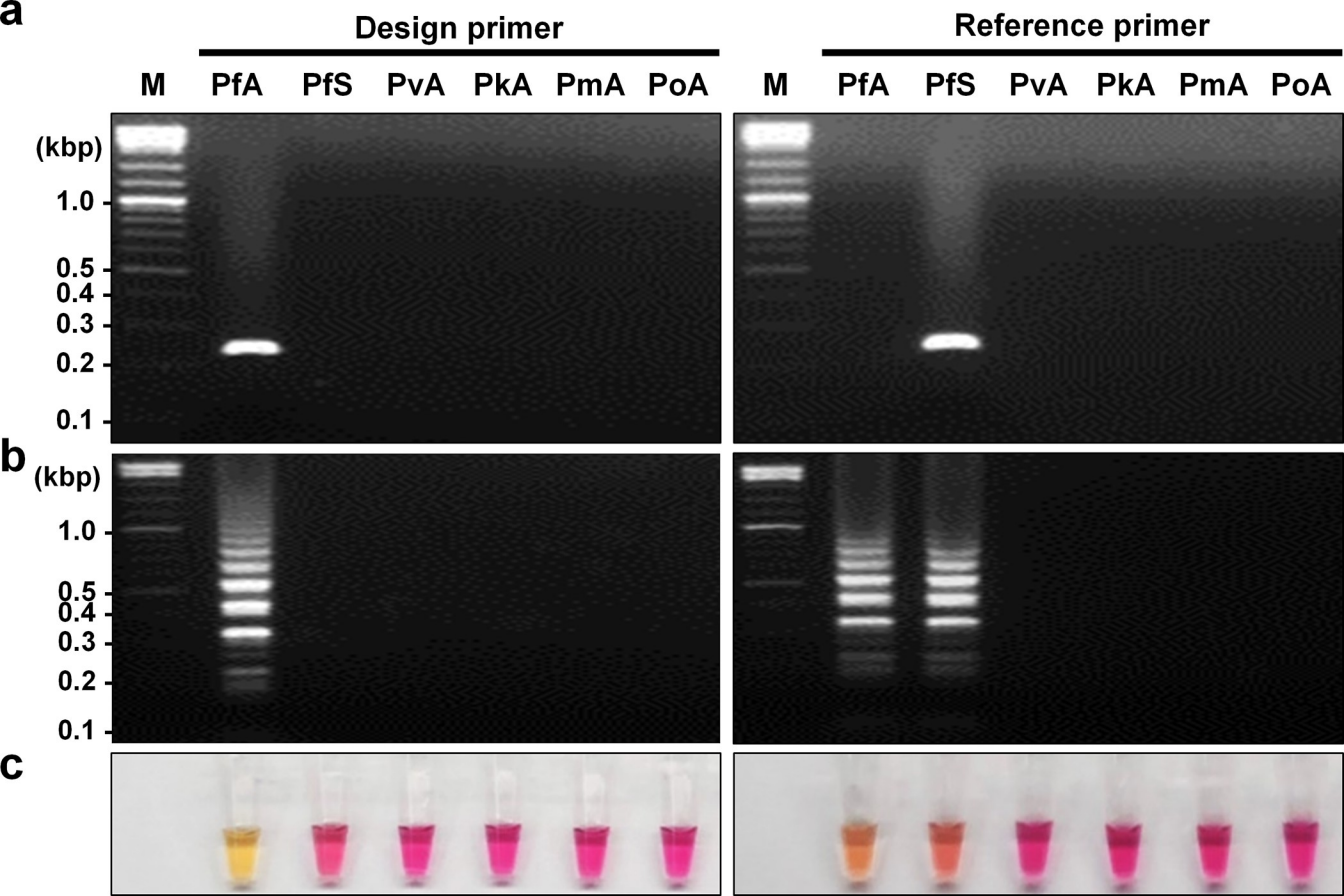
The cLAMP specificity of the design (left panel) and reference (right panel) primer set. The plasmid DNA (pDNA) containing *18S rRNA* genes from both the *P*. *falciparum* S-type (PfS) and A-type (PfA) were utilized to confirm primer specificity. A-type *18S rRNA* genes from other *Plasmodium* species were also employed for confirmation: *P*. *vivax* (PvA), *P*. *knowlesi* (PkA), *P*. *malariae* (PmA), and *P*. *ovale* (PoA). **(a)** The results indicated the specificity of PCR with the outer primer set (F3-B3c) for their corresponding fragments. PfA, with the designed primer, exhibited an expected size of 229 bp, while PfS, with the reference primer, showed an expected size of 220 bp. Lane M represents the DNA ladder mix. **(b)** The cLAMP results were visualized through 2% agarose gel electrophoresis. **(c)** The cLAMP results corresponded to (b) and were confirmed through naked eye direct observation. Yellow/orange coloration indicated a positive, whereas pink indicated a negative.

PCR was conducted to confirm the species-specificity of each outer primer set (F3-B3c). The designed and reference outer primers showed specific amplification in the A-type (PfA) and S-type (PfS) *18S rRNA* genes of *P*. *falciparum*, with estimated amplicon sizes of 229 bp and 220 bp, respectively **(Fig 2A)**. After confirming the specificity of the outer primer set, the cLAMP was conducted under optimal conditions at 65°C for a 30-minute incubation. The presence of the cLAMP amplicon was confirmed by visualizing on a 2% agarose gel electrophoresis, showing a typical ladder pattern of LAMP reaction **(Fig 2B)**. The designed primer showed specific amplification in the A-type of *18S rRNA* (PfA). In contrast, the reference primer set amplified both the A-type and S-type of *18S rRNA* (PfA and PfS) **(Fig 2B)**. The direct observation by naked eye of the designed primer showed an obvious color change in PfA to yellow, while the reference primer showed intermediate color changes in both PfA and PfS **(Fig 2C)**. Taken together, the cLAMP primers showed no cross-amplification issues with other *Plasmodium* species.

### Analytical Limit of detection (LoD) of cLAMP

The accurate LoD of cLAMP was estimated by two methods utilizing synthesized *18S rRNA* genes to calculate the target gene copy number and *in vitro* cultured *P*. *falciparum*.

**Fig 3:**
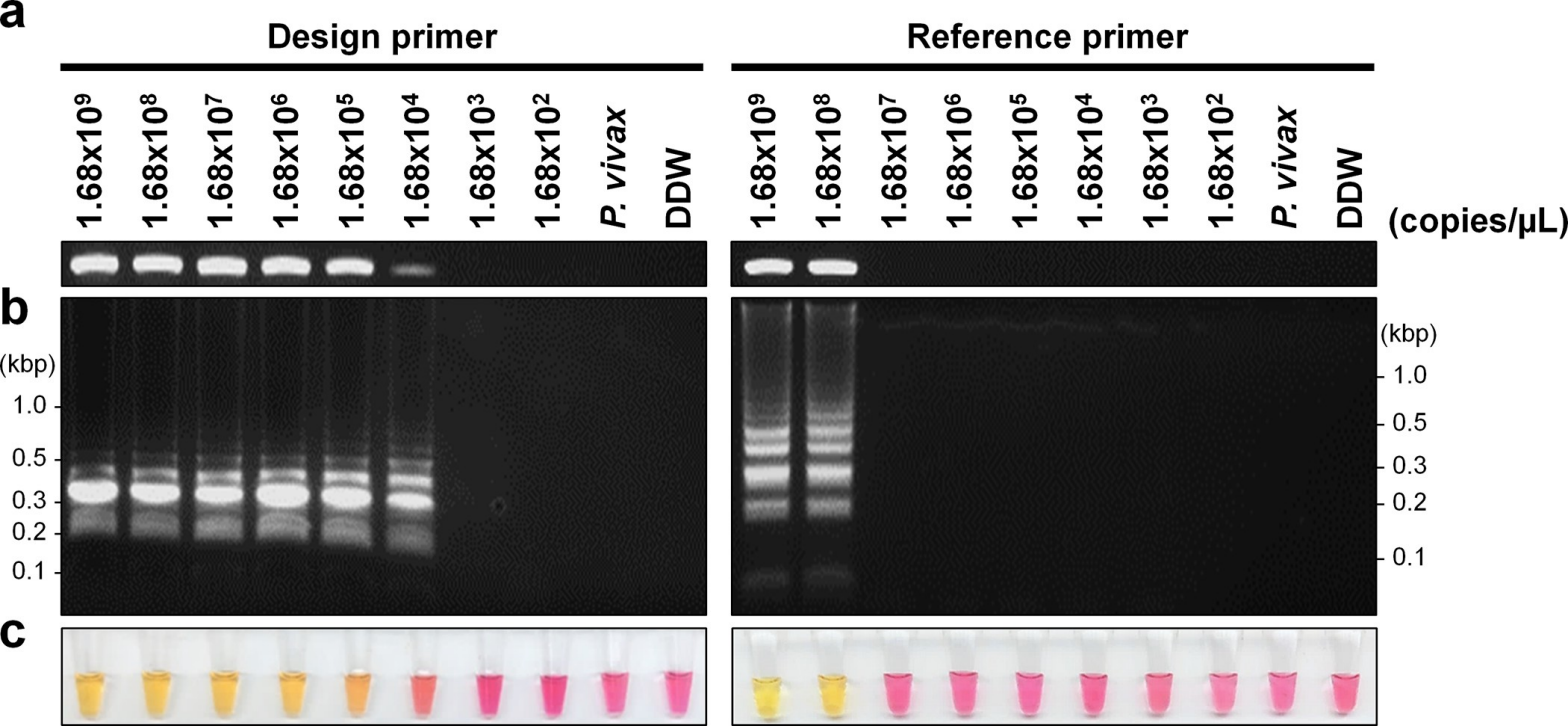
Limit of detection based on plasmid DNA. **(a)** PCR detection using the outer primer set with serially diluted plasmid DNA. Ten microliters of the PCR products were visualized on a 2% agarose gel. **(b)** One microliter of the endpoint products from the cLAMP reaction was observed on a 2% agarose gel. **(c)** cLAMP results were interpreted by the naked eye; yellow/orange indicates a positive result, whereas pink indicates a negative result. The lane contained *P*. *vivax* gDNA (*P*. *vivax*) and DDW, which were used as negative controls.

For the determination of the LoD based on the target gene copy number, synthesized *18S rRNA* genes were employed. The copy number of the A-type *18S rRNA* was estimated to be 1 x 10^4^ copies per ring stage parasite in a previous study [32]. The PfA and PfS were serially diluted ten-fold from 1.68 x 10^9^ copies/μL which corresponding to 168,000 ring stage parasites/μL. The PCR using outer primers (F3-B3c) detection limit estimated a concentration of 1.68 x 10^4^ copies/μL (corresponding to 1.68 ring stage parasites/μL) when using the designed primer and 1.68 x 10^8^ copies/μL with the reference primer **(Fig 3A)**. The cLAMP results were confirmed through electrophoresis and demonstrated the same sensitivity as the PCR results **(Fig 3B)**. The LoD of the designed primer was 10^4^ times higher in sensitivity compared to the reference primer in both PCR and cLAMP. However, when observed with the naked eye, the designed primer exhibited an intermediate color change at the endpoint of LoD (1.68 x 10^4^ copies/μL) **(Fig 3C)**.

**Fig 4:**
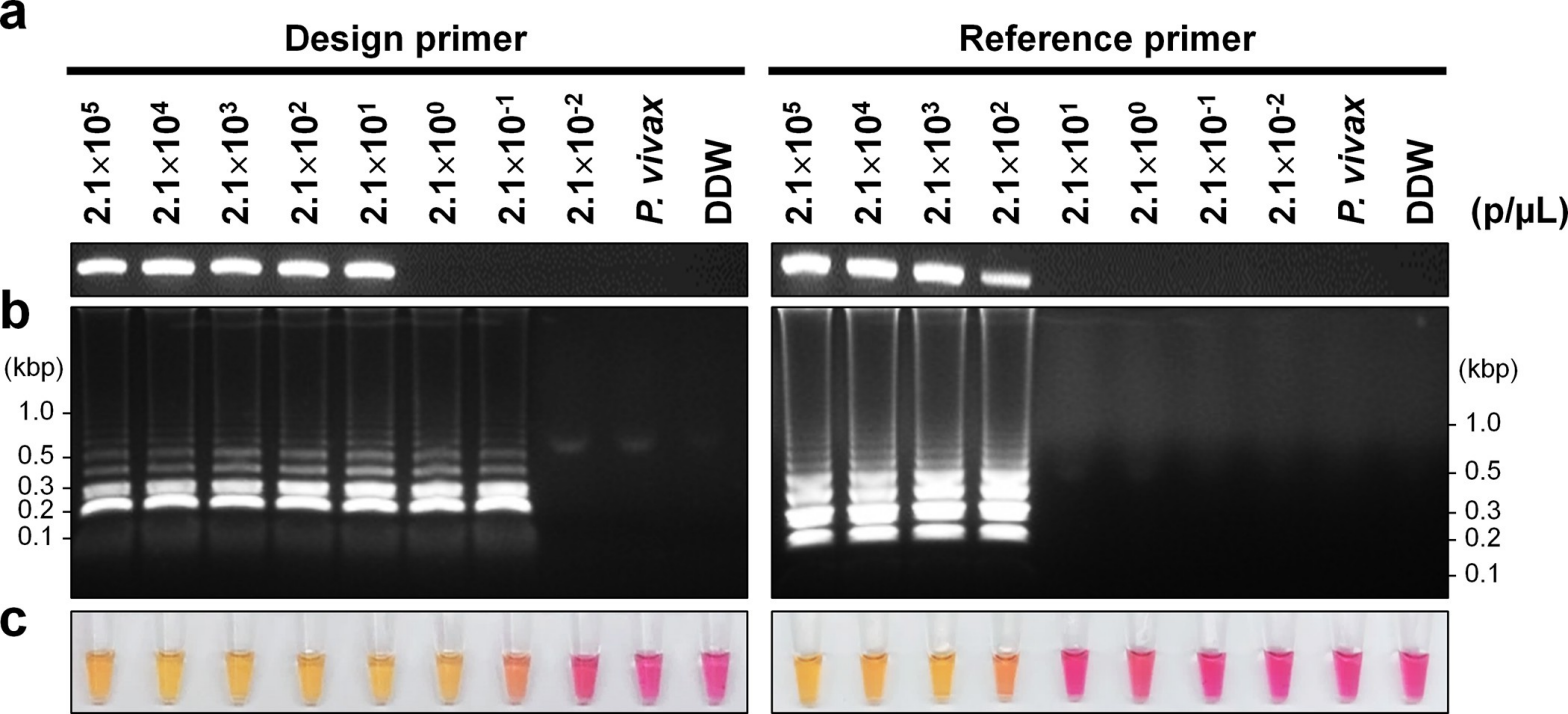
Limit of detection test based on parasite count. **(a)** PCR detection using the outer primer set with serially diluted parasites genomic DNA (parasites/μL; p/μL). Ten microliters of the amplicon were visualized on a 2% agarose gel. **(b)** One microliter of the cLAMP product was visualized on a 2% agarose gel. Negative control consisting of *P*. *vivax* gDNA (*P*. *vivax*) and DDW were used to confirm specificity. **(c)** The cLAMP results corresponding to (b) were visualized with the naked eye; yellow indicated a positive, whereas pink indicated a negative.

The LoD based on parasite count (parasites/μL) was performed using genomic DNA extracted from cultured *P*. *falciparum*. Initially, PCR was performed to confirm the LoD of both the design and reference outer primers. The designed primer had a 10 times higher amplification efficiency, resulting in a detection limit of 21 parasites/μL, while the reference primer had a detection limit of 210 parasites/μL **(Fig 4A)**. When compared with PCR result, the cLAMP with the designed primer showed a 100-fold higher sensitivity in detecting up to 0.21 parasites/μL **(Fig 4B)**. In contrast, the reference primer showed the same LoD in both PCR and cLAMP **(Fig 4B)**. When comparing the cLAMP results between the designed and reference primers, it revealed a 1,000-fold higher sensitivity in the designed primer **(Fig 4C)**. However, when observed with the naked eye, both the designed and reference primer showed an intermediate color change at each endpoint of LoD 0.21 parasites/μL and 210 parasites/μL, respectively **(Fig 4C)**. This result showed that cLAMP has a higher accuracy and more sensitivity in detecting low parasite numbers compared to PCR.

### Performance of the cLAMP

**Fig 5:**
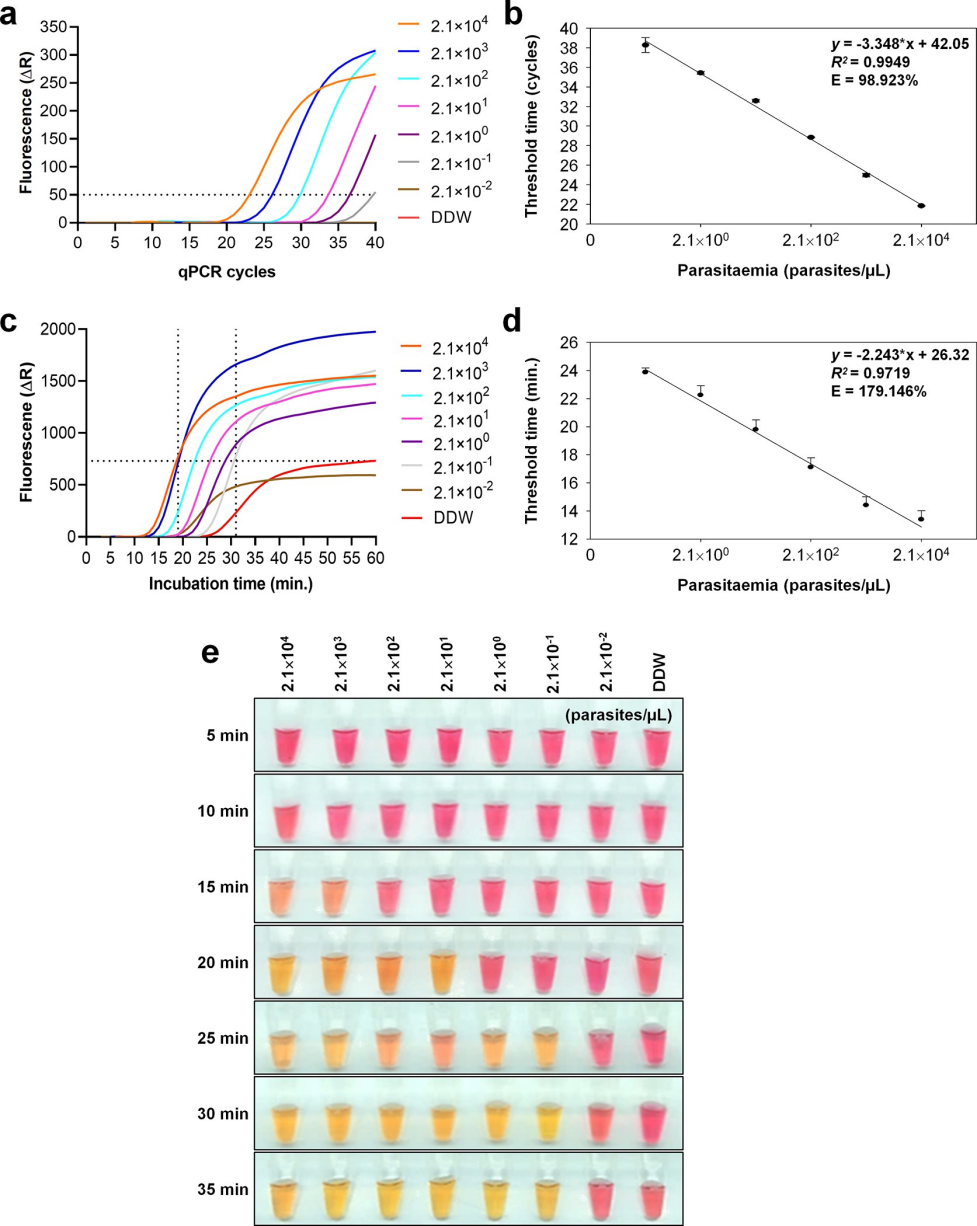
The performance verification of cLAMP. **(a)** Fluorescence (ΔR) detection was based on ten-fold diluted *P*. *falciparum* 3D7 gDNA (from 2.1 x 10^4^ to 2.1 x 10^−2^ parasites/μL) using qPCR. The dotted line indicates the analytical positive threshold line (ΔR = 50). **(b)** The comparison of parasite count (parasites/μL) versus qPCR cycles shows a linear regression fit with *r*^2^ = 0.9949. All error bars represent the standard deviation of triplicate reactions. **(c)** RT-cLAMP detection results were acquired using SYBR green signal every minute of incubation time. The horizontal dotted line indicates the analytical positive threshold line (ΔR = 730), while the vertical dotted line indicates the time point of positive reading for both 2.1 x 10^4^ and 10^−1^ parasites/μL. **(d)** The comparison of parasite count versus RT-cLAMP incubation time (min.) shows a linear regression fit with *r*^2^ = 0.9719. All error bars represent the standard deviation of triplicate reactions. **(e)** Observation of color changes by the naked eye was done every 5 minutes; yellow indicates a positive, whereas pink indicates a negative.

To confirm the cLAMP performance, a real-time cLAMP (RT-cLAMP) was conducted and compared to quantitative PCR (qPCR) diagnosis methods [11]. Based on the analytical threshold (ΔR = 50), the qPCR positive read was determined at 21,000 parasites/μL from 23 PCR cycles **(Fig 5A)**. A plot correlating the log threshold cycle with log parasite count (parasites/μL) showed a strong linear regression (*r*^*2*^ = 0.9949) in qPCR detection, with 98.9% amplification efficiency, indicating a 1.989-fold amplification per cycle **(Fig 5B)**.

To estimate the amount of amplicon in RT-cLAMP, fluorescence data were acquired at 1-minute intervals for up to 1 hour. The fluorescence signal surpassed the analytical positive threshold (ΔR = 730) after 19 minutes of incubation at a parasite count of 21,000 parasites/μL. **(Fig 5C)**. The lowest detection was observed at 0.21 parasites/μL after 31 minutes of incubation, which was consistent with the LoD of qPCR **(Fig 5A and 5C)**. A plot correlating the log threshold with time in minutes versus log parasite count revealed a linear regression (*r*^2^ = 0.9719) with 179.1% amplification efficiency, indicating a 2.791-fold amplification per minute **(Fig 5D)**.

The naked eye observation of the cLAMP showed distinguishable color changes after 15 minutes of incubation at 21,000 parasites/μL **(Fig 5E)**. The lowest detection of 0.21 parasites/μL showed a color change at the 25-minute time point **(Fig 5E)**. However, parasite counts lower than the lowest detection limit (0.021 parasites/μL) were not detectable by RT-cLAMP and naked eye observation, consistent with the sensitivity of qPCR **(Fig 5C and 5E)**. Taken together, the cLAMP analytical LoD was determined to be 0.21 parasites/μL, and it was detectable within 31 minutes in both the RT-cLAMP and 25 minutes by the naked eye observation.

### The sensitivity and specificity of cLAMP diagnosis using clinical specimens

**Fig 6:**
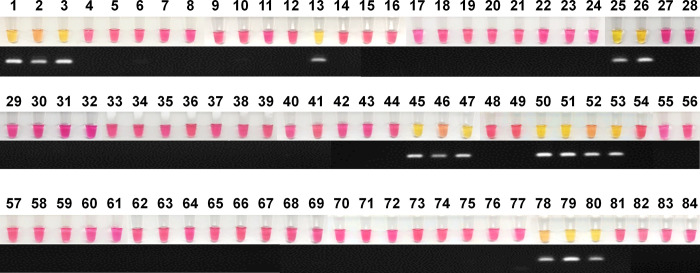
Validation of cLAMP using 84 clinical specimens. Comparison of diagnosis performance between cLAMP and nPCR for *P*. *falciparum* detection. The cLAMP reaction was read by the naked eye; yellow/orange indicates a positive result, and pink indicates a negative result. The nPCR result was visualized on a 2% agarose gel.

To assess the performance comparison of cLAMP, we conducted and compared three different molecular diagnostic tests: nested PCR (nPCR), quantitative PCR (qPCR), and colorimetric LAMP (cLAMP). The molecular detection was carried out as blind tests, with unknown positivity in microscopic examination. The direct comparisons between cLAMP and nPCR are shown in **Fig 6A**. The determination of *P*. *falciparum*-positive isolates was based on microscopic examination results, allowing us to calculate the sensitivity and specificity of each molecular diagnostic method. Based on the microscopic examination, 16 out of the 84 isolates were positive for *P*. *falciparum*, and all three molecular diagnostic methods successfully detected the positive samples **(S3 Table)**. The sixteen microscopy-positive samples were identified with a range of 1,069 to 285,290 parasites/μL (mean = 63,114 parasites/μL) **(S3 Table)**. The results for nPCR, qPCR, and cLAMP revealed sensitivity and positive predictive values of 100% (95% CI: 79.41–100%) **(Table 2)**. Also, specificity and negative predictive values showed to be 100% (95% CI: 94.72–100%) **(Table 2)**. The overall accuracy reached up to 100% (95% CI: 95.70–100%) in all tests when compared with microscopic examination **(Table 2)**.

**Table 2:** Performance comparison between microscopy versus each molecular diagnosis methods. Sensitivity, specificity, positive predictive value, negative predictive value, and accuracy were calculated based on the 84 isolates. The number of isolates used was 16 true positive (ranging in 1,069~285,290 paraistes/μL) and 68 true negative based on microscopic examination result.

Parameters	Nested PCR	Quantitative PCR	Colorimetric LAMP
**Sensitivity** (95% CI*)	100% (79.41%~100%)	100% (79.41%~100%)	100% (79.41%~100%)
**Specificity** **(95% CI)**	100% (94.72%~100%)	100% (94.72%~100%)	100% (94.72%~100%)
**Positive Predictive Value (95% CI)**	100% (79.41%~100%)	100% (79.41%~100%)	100% (79.41%~100%)
**Negative Predictive Value (95% CI)**	100% (94.72%~100%)	100% (94.72%~100%)	100% (94.72%~100%)
**Accuracy** **(95% CI)**	100% (95.70%~100%)	100% (95.70%~100%)	100% (95.70%~100%)

*CI: Confidence interval.

## Discussion

Early diagnosis is the most important strategy for malaria control in hyperendemic areas. However, the absence of affordable healthcare options and accurate tools for parasite early detection is the challenge to malaria eradication in resource-limited areas [33]. In the early 2000s, LAMP emerged as a novel amplification method with superior properties to be used in field studies. Although most LAMP assays were conducted in a laboratory settings, their applicability in the field is facilitated by cost-effectiveness, easy to performance, and rapidity. Furthermore, LAMP results can be visually interpreted through turbidimetry, fluorescence, or colorimetric changes under a consistent incubation temperature that enhanced their field application [29,34–36]. Thus, this study focused on developing a sensitive colorimetric LAMP (cLAMP) primer set and its assay for the early detection of *P*. *falciparum* at low parasitemia, potentially enhancing its utilization in the field.

The first study of LAMP assay for *P*. *falciparum* achieved 95% sensitivity and 99% specificity when compared to PCR [37]. Since then, several studies have been conducted to enhance the efficiency of LAMP diagnosis. Among those, some studies have targeted the S-type of the *18S rRNA* genes for parasite detection [15,38]. However, the S-types of the *18S rRNA* were found to be abundant in the gametocyte for S1-type and in sporozoites within the mosquito salivary gland for S2-type [30]. Besides, variation between S1-type and S2-type has been observed in *P*. *falciparum*, *P*. *berghei*, and *P*. *yoelii* [30]. Hence, the primer set designed in this study targeted the A-type of the *18S rRNA* gene for dual gene targeting, which shares nearly 100% sequence identity on chromosomes 5 and 7 [30]. One study using patient whole blood demonstrated that 96.8% patients presented higher copy numbers of the A-type *18S rRNA* gene than S-type *18S rRNA* gene in the erythrocytic stage [39]. Further, the A-type of *18S rRNA* gene is stable and is expressed at high levels with approximately 1 x 10^4^ copies in the asexual stage, particularly in the ring stage of parasites [32,40,41]. When collecting peripheral blood from *P*. *falciparum*-infected patients for diagnosis, it typically contains only ring stage and gametocyte forms. Therefore, selecting the A-type of the *18S rRNA* gene is the most suitable target for early detection, especially in cases of low parasitemia.

In this study, the performance of cLAMP was demonstrated at a LoD of 0.21 parasites/μL, whereas several studies have reported LoDs ranging from 1 to 5 parasites/μL achieved using various mitochondrial DNA targets [25,28,42]. The sensitivity and specificity reported in previous studies ranged from 95% to 98.5% and 96% to 100%, respectively, and all of these studies demonstrated high-quality performance [15,37,42]. However, the reported studies required a reaction incubation time of 45 minutes to 2 hours and the result reading necessitated the use UV illuminator or bioluminescent real-time reporter system, which pose difficulties in field utilization. An important feature of cLAMP assay in this study is its ability to swiftly detected low parasite levels in a shorter incubation time, without the need for specialized equipment [22]. Furthermore, colorimetric detection methods are able to prevent cross-contamination issues when applied in the field condition [23].

Although this study demonstrated many advantages, it is also compromised with certain limitations. Sample preparation needs to be considered, as it can be challenging to conduct in the field. Many methods have been developed to simplify genomic DNA extraction [43,44]. Thus, this cLAMP assay needs to be combined with a simplified genomic DNA extraction system for field application. In this study, we used dried blood spots for the extraction of genomic DNA in the laboratory. The dried blood spot contain various biologically active substances, including cellular components and enzyme inhibitors, which can potentially affect the diagnostic sensitivity when compared to fresh blood samples [45]. The lowest parasite count in the specimen pool of this study was 1,069 parasites/μL (0.02% parasitemia), successfully detected by cLAMP. However, the tested sample pool did not contain an extremely low parasitemia, which can be detected only by molecular detection method and not by microscopy. Thus, this could pose a limitation for statistical analysis. This study used a limited number of clinical isolates, and it is necessary to employ a more varied range of sample pools, including those with fresh specimens and extremely low parasitemia. Thus, it requires confirmation under various sample conditions in the further. In addition, similar to previous studies, interpreting intermediate color changes can be affected by individual differences which is also considered a significant limitation in cLAMP diagnosis for making definitive decisions regarding *P*. *falciparum* infections, especially when dealing with extremely low parasitaemia (<0.21 parasites/μL) [22,29]. Such suspected results need to be reconfirmed by the other molecular detection technique.

Despite these limitations, the cLAMP with a newly designed primer set demonstrated higher sensitivity, faster performance, and ease of use compared to other molecular detection methods. These findings offer a time effective technique and simpler result interpretation, making cLAMP a promising choice for the point-of-care (PoC) tool for early malaria detection and large-scale screening.

## Supporting information

S1 Fig The alignment of the primer binding regions within the human malaria parasites. Two *18S rRNA* sequences on chromosome 5 (accession no. XR_002273101.1) and chromosome 7 (accession no. XR_002273081.2) are indicated by different color arrows for each primer binding region (black for F3/B3c pair, green for F2/B2c, yellow for F1c/B1, and red for LpF/LpB).
(DOCX)

S1 Table The partial species-specific nucleotide sequences of the 18S rRNA genes from five human malaria parasites: *P*. *falciparum*, *P*. *vivax*, *P*. *malariae*, *P*. *knowlesi*, *and P*. *ovale*. (DOCX)

S2 Table Primer sequences used in PCR, qPCR, and nPCR for *P*. *falciparum* detection. (DOCX)

S3 Table A summary of *P*. *falciparum* positive isolates in each diagnosis method. (DOCX)
