# Supplementary material for: Enhancing malaria detection in resource-limited areas: A high-performance colorimetric LAMP assay for Plasmodium falciparum screening
Source: PLoS One. 2024 Feb 9;19(2):e0298087. doi: 10.1371/journal.pone.0298087 (PMC10857711; doi:10.1371/journal.pone.0298087)
Supplement: S1 Fig — Two 18S rRNA sequences on chromosome 5 (accession no. XR_002273101.1) and chromosome 7 (accession no. XR_002273081.2) are indicated by different color arrows for each primer binding region (black for F3/B3c pair, green for F2/B2c, yellow for F1c/B1, and red for LpF/LpB). (DOCX) [file pone.0298087.s001.docx]

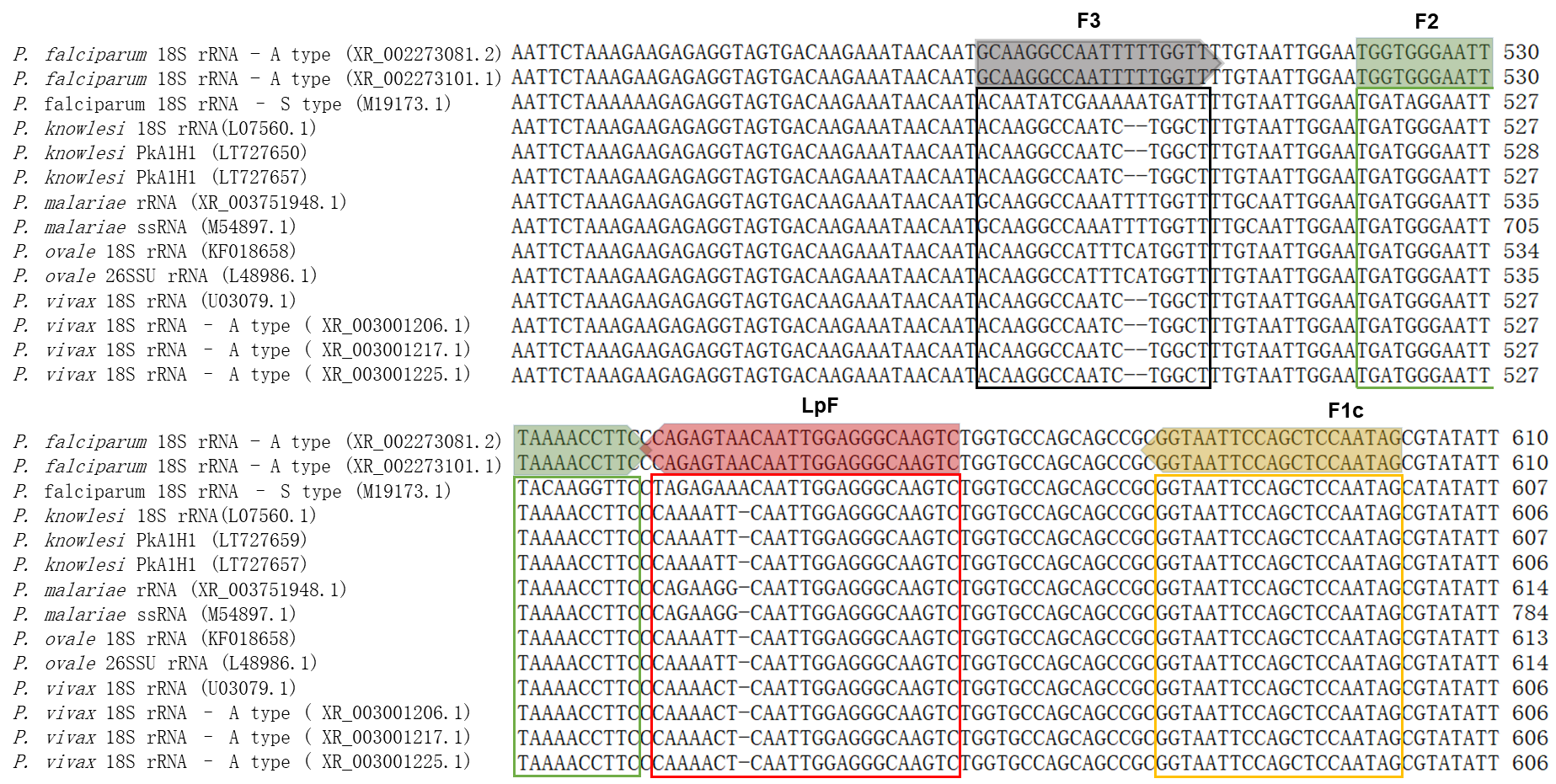


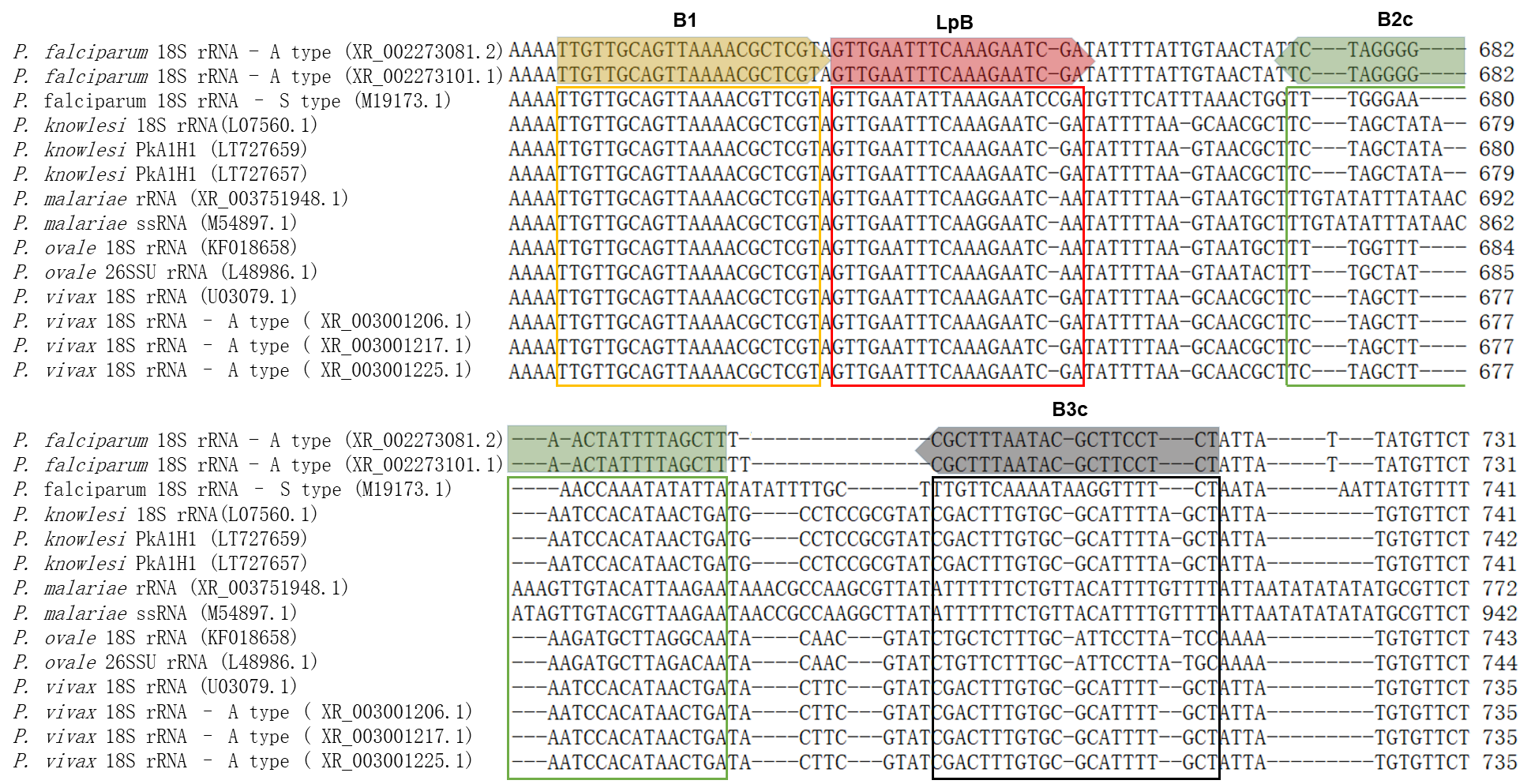


**S1 Fig.** **The alignment of the primer binding regions within the human malaria parasites.** Two *18S rRNA* sequences on chromosome 5 (accession no. XR_002273101.1) and chromosome 7 (accession no. XR_002273081.2) are indicated by different color arrows for each primer binding region (black for F3/B3c pair, green for F2/B2c, yellow for F1c/B1, and red for LpF/LpB).
