# Supplementary material for: Enhancing malaria detection in resource-limited areas: A high-performance colorimetric LAMP assay for Plasmodium falciparum screening
Source: PLoS One. 2024 Feb 9;19(2):e0298087. doi: 10.1371/journal.pone.0298087 (PMC10857711; doi:10.1371/journal.pone.0298087)
Supplement: S1 Table — (DOCX) [file pone.0298087.s002.docx]

**S1 Table. The partial species-specific nucleotide sequences of the 18S rRNA genes from five human malaria parasites*: P. falciparum, P. vivax, P. malariae, P. knowlesi, and P. ovale*.**

| Label | Assession number | Sequence (5’-3’) |
| --- | --- | --- |
| *Plasmodium falciparum* | | |
| PfA | *XR_002273101.1*  *XR_002273081.2* | ATAACAATGCAAGGCCAATTTTTGGTTTTGTAATTGGAATGGTGGGAATTTAAAACCTTCCCAGAGTAACAATTGGAGGGCAAGTCTGGTGCCAGCAGCCGCGGTAATTCCAGCTCCAATAGCGTATATTAAAATTGTTGCAGTTAAAACGCTCGTAGTTGAATTTCAAAGAATCGATATTTTATTGTAACTATTCTAGGGGAACTATTTTAGCTTTCGCTTTAATACGCTTCCTCTATTATTATGTTCTTTAAATAACAAAGATTCTTTTTAAAATCCCCACTTTTGCTTTTGCTTTTT |
| PfS | *M19173.1* | GCAGCAGGCGCGTAAATTACCCAATTCTAAAAAAGAGAGGTAGTGACAAGAAATAACAATACAATATCGAAAAATGATTTTGTAATTGGAATGATAGGAATTTACAAGGTTCCTAGAGAAACAATTGGAGGGCAAGTCTGGTGCCAGCAGCCGCGGTAATTCCAGCTCCAATAGCATATATTAAAATTGTTGCAGTTAAAACGTTCGTAGTTGAATATTAAAGAATCCGATGTTTCATTTAAACTGGTTTGGGAAAACCAAATATATTATATATTTTGCTTTGTTCAAAATAAGGTTTTC |
| *Plasmodium vivax* | | |
| PvA | *U03079* | CAAGGCCAATCTGGCTTTGTAATTGGAATGATGGGAATTTAAAACCTTCCCAAAACTCAATTGGAGGGCAAGTCTGGTGCCAGCAGCCGCGGTAATTCCAGCTCCAATAGCGTATATTAAAATTGTTGCAGTTAAAACGCTCGTAGTTGAATTTCAAAGAATCGATATTTTAAGCAACGCTTCTAGCTTAATCCACATAACTGATACTTCGTATCGACTTTGTGCGCATTTTGCTATTATGTGTTCTTTTAATTAAAATGATTCTTTTTAAGGACTTTCTTTGCTTCGGCTTGGAAGTCC |
| *Plasmodium knowlesi* | | |
| PkA | *L07560.1* | TATGAATCTGTACAGTGTTGATAAGGAACGGATTGTCTTGCCAAGGATATTTATCTCCACCGATAAGGAGAGTATCAAATGTCCATGTGAGCCTGAACACATTTCCAATAGTACCTGCAACTTTTACGTTTGTAACTGTGTAGAGAAGAGAGCAGAAATTAAGGAAAATAACGAAGTTATCATAAAGGAAGAATTTAAGGAGGATTATGAAAATCCGGACGGCAAACATAAGAAGAAGATGCTACTAATTATTATCGGAGTAACTGGAGCTGTTTGTGTCGTCGCAGTAGCCTCCTTGTT |
| *Plasmodium malariae* | | |
| PmA | *M54897* | AGGTAGTGACAAGAAATAACAATGCAAGGCCAAATTTTGGTTTTGCAATTGGAATGATGGGAATTTAAAACCTTCCCAGAAGGCAATTGGAGGGCAAGTCTGGTGCCAGCAGCCGCGGTAATTCCAGCTCCAATAGCGTATATTAAAATTGTTGCAGTTAAAACGCTCGTAGTTGAATTTCAAGGAATCAATATTTTAAGTAATGCTTTGTATATTTATAACATAGTTGTACGTTAAGAATAACCGCCAAGGCTTATATTTTTTCTGTTACATTTTGTTTTATTAATATATATATGCGTT |
| *Plasmodium ovale* | | |
| PoA | *L48986.1* | AAGAAATAACAATACAAGGCCATTTCATGGTTTTGTAATTGGAATGATGGGAATTTAAAACCTTCCCAAAATTCAATTGGAGGGCAAGTCTGGTGCCAGCAGCCGCGGTAATTCCAGCTCCAATAGCGTATATTAAAATTGTTGCAGTTAAAACGCTCGTAGTTGAATTTCAAAGAATCAATATTTTAAGTAATACTTTTGCTATAAGATGCTTAGACAATACAACGTATCTGTTCTTTGCATTCCTTATGCAAAATGTGTTCTTATTATAAAAAGGATTCTTTTTAAAATCTCTTTTGC |
