# Supplementary material for: Enhancing malaria detection in resource-limited areas: A high-performance colorimetric LAMP assay for Plasmodium falciparum screening
Source: PLoS One. 2024 Feb 9;19(2):e0298087. doi: 10.1371/journal.pone.0298087 (PMC10857711; doi:10.1371/journal.pone.0298087)
Supplement: S2 Table — (DOCX) [file pone.0298087.s003.docx]

**S2 Table. Primer sequences used in PCR, qPCR, and nPCR for *P. falciparum* detection.**

| Primers | | Sequences (5’-3’) | Length (mer) | Reference |
| --- | --- | --- | --- | --- |
| Conventional PCR | | | |  |
| Control primer | F3 | TGTAATTGGAATGATAGGAATTTA | 24 | [15] |
|  | B3c | GAAAACCTTATTTTGAACAAAGC | 23 | [15] |
| Design primer | F3 | GCAAGGCCAATTTTTGGTT | 19 | This study |
|  | B3c | AGAGGAAGCGTATTAAAGCG | 21 | This study |
| Nested PCR | |  |  |  |
| rPLU1_F | | TCAAAGATTAAGCCATGCAAGTGA | 24 | [10] |
| rPLU5_R | | CCTGTTGTTGCCTAAAACTTC | 21 | [10] |
| rFAL1_F | | TTAAACTGGTTTGGGAAAACCAAATATATT | 30 | [10] |
| rFAL2_R | | ACACAATGAACTCAATCATGACTACCCGTC | 30 | [10] |
| Quantitative PCR | |  |  |  |
| Pf 18S rRNA forward | | ATTGCTTTTGAGAGGTTTTGTTACTTT | 27 | [11] |
| Pf 18S rRNA reverse | | GCTGTAGTATTCAAACACAATGAACTCAA | 29 | [11] |
| Pf Probe (FAM) | | CATAACAGACGGGTAGTCAT | 20 | [11] |
