# Supplementary material for: Enhancing malaria detection in resource-limited areas: A high-performance colorimetric LAMP assay for Plasmodium falciparum screening
Source: PLoS One. 2024 Feb 9;19(2):e0298087. doi: 10.1371/journal.pone.0298087 (PMC10857711; doi:10.1371/journal.pone.0298087)
Supplement: S3 Table — (DOCX) [file pone.0298087.s004.docx]

**S3 Table. A summary of *P. falciparum* positive isolates in each diagnosis method.**

| **No.** | **Microscopy**  **(p/μL)** | **nPCR**  **(+/-)** | **qPCR**  **(Cq)** | **cLAMP**  **(+/I*/-)** |
| --- | --- | --- | --- | --- |
| 1 | 22,010 | + | 25.66 | + |
| 2 | 1,069 | + | 32.15 | I |
| 3 | 285,290 | + | 23.09 | + |
| 13 | 62,000 | + | 24.91 | + |
| 25 | 66,242 | + | 24.75 | + |
| 26 | 54,745 | + | 24.12 | + |
| 45 | 50,526 | + | 26.56 | + |
| 46 | 69,742 | + | 25.44 | I |
| 47 | 92,504 | + | 24.99 | + |
| 50 | 28,317 | + | 27.64 | + |
| 51 | 28,856 | + | 28.03 | + |
| 52 | 4,966 | + | 28.89 | I |
| 53 | 152,381 | + | 24.18 | + |
| 78 | 10,628 | + | 29.65 | + |
| 79 | 13,423 | + | 28.01 | + |
| 80 | 67,126 | + | 26.20 | + |

*I: intermediate color change, p/μL: parasites/μL, +: positive, -: negative
